# Breast cancer revealed by a paraneoplastic cerebellar syndrome: about one case and literature review

**DOI:** 10.11604/pamj.2015.22.25.6217

**Published:** 2015-09-10

**Authors:** Dembélé Adama, Bambara Moussa, Macoumi Emmanuel, Ullmann Dennis

**Affiliations:** 1 Department of Obstetrics, Gynecology and Reproductive Medicine, Souro Sanou Teaching University, Hospital of Bobo Dioulasso, Burkina Faso; 2 Higher Institute of Health Sciences, Polytechnic University of Bobo Dioulasso, Burkina Faso; 3 Louis Pasteur Hospital Center, Maternity Service, Cherbourg, France; 4 Training and Research Unit in Health Sciences, University of Ouagadougou, Ouagadougou, Burkina Faso

**Keywords:** Breast cancer, early diagnosis, paraneoplastic cerebellar syndrome, neural antibodies, anti-Yo, autoimmunity

## Abstract

To describe a case of breast cancer manifested by cerebellar syndrome and to establish a relationship between breast cancer and Paraneoplastic syndromes through the presence of anti- yo antibodies in serum and cerebrospinal fluid of a patient. Our patient was 52 years old, Multipara with 5 children alive. She had been 3 years post-menopausal under Hormonal Replacement Therapy. Weight: 46.7 Kg; Height: 1.60 m; Body Surface Area: 1.59 m^2^. Nil history of alcohol or tobacco smoking. Nilhistory suggestive of malignancies or autoimmune diseases. Her Blood group was oRhpositive, nil presence of irregular agglutinins. She was admitted to the neurology service for vertigo and it was determined an isolated cerebellar syndrome. All tests were negative including tumor markers and radiological imaging. The clinical gynecological examination was perfectly normal. The diagnosis hypothesis was “meningo-encephalocerebellitis of viral origin” but with persistence and aggravation of the cerebellar syndrome, despite treatment. We decided to search, antibodies, anti-Hu, anti-Yo, anti-Ri, and anti Ta. Anti Yo was positive + + + in the cerebrospinal fluid and serum of the patient. The search for a gynecological cancer included a mammography which revealed micro calcifications in the left breast + + +. A lumpectomy of the left breast accompanied with x-ray identification of the micro calcifications was done and the histology showed a High Grade Intraductal carcinoma of the left breast with two homes of 3mm and 1 mm, corresponding to an infiltrating carcinoma of the left breast, grade II tumor of Scarff and Bloom (SBRII, 21 N + / 26, RH +, low Ki 67) and Estrogen and progesterone receptor positive +: multifocal cancer. Following the lumpectomy, mastectomy withganglion clearing was done with adjuvant chemotherapy (FEC 6 Cycles): histology still showed Infiltrating Intraductal Carcinoma of the left breast, grade II tumor of Scarff and Bloom. Radiotherapy was followed and he patient was placed on hormonal therapy with Tamoxifen. The Patient's general condition was good with regression of cerebellar syndrome. Anti-Yo auto antibodies are quasi-specific for gynecological or breast tumors. Several hypotheseshave been advanced on the pathophysiology and one wonders if someday, it will fail to do a very early diagnosis of cancer, including the breast cancers on the basis of the antigen-antibody reaction.

## Introduction

The early diagnosis of breast cancer allows for better care and support of patients [1]. Paraneoplastic syndromes are heterogeneous manifestations, caused by malignant tumors that may be associated with breast cancer. Clinical signs that constitute these syndromes may be neurological, hematological, dermatological, endocrinological, renal, vascular or rheumatological according to the tumors [2]. Paraneoplastic Cerebellar Syndrome (PCS), when associated with cancer of the breast in a patient, comes with presence of anti-neuronal antibodies in the serum of the patient before its discovery in 65% of cases [3]. It could be a future element in the early diagnosis of breast cancer. This is a casereport study of the evolution of cerebellar syndrome in a patient. Investigations for the aetiologic research of cerebellar syndrome led to laboratory assays for anti-neuronal specific antibodies in the patient's serum and cerebrospinal fluid. The presence of these antibodies raised a high suspicion of a gynecologic cancer in the patient. The diagnostic approach revealed leftbreast pathology and allowed for an early clinical management of the patient. The laboratory assays for specific anti- neuronal antibodies took place at the “Hospital de La Pitié Salpêtrière” and the surgical and medical care of the patient took place at the “Centre Hospitalier Louis Pasteur” of Cherbourg in France.

## Patient and observation

The patient is 52 year-old, weighed 46.7 Kg with height measuring 1, 60 m. She has a body surface area of 1.59 m^2^. She hails from North Africa. She is a grand multipara with 5 children Alive. She neither smokes nor drinks alcohol. There is no family history suggestive of autoimmunediseases, genital cancers, and other malignancies. She is Blood group oRh positive with nil presence of irregular agglutinins. She developed Hashimoto thyroiditis since 1995 and is on Levothyrox and Cortancyl. She is also taking beta blockers for hypertension with arrhythmias. She is three years post- menopausal and under Hormonal Replacement Therapy. She was admitted in the Department of Neurology of the Centre Hospitalier de Cherbourg for vertigo. Clinical examination revealed an isolated cerebellar syndrome with positive Romberg, positive Finger-Nose Test, and horizontal nystagmus on lateral gaze. All tests for aetiology were negative including tumor markers and radiological imaging. Clinical gynecologicalexamination was normal. The initial diagnostic hypothesis made was a Meningo-encephalocerebellitis of viral origin. There waspersistence and even aggravation of the cerebellar syndrome despite all the treatments. It was decided that a specific determination of auto neuronal antibodies be made: searching antibodies anti-Hu, anti- Yo, anti-Ri, anti- Ta. There was a resultant positivity for anti Yo antibody in the cerebral spinal fluid and serum of the patient. Based on this fact the search for a gynecologic cancer had been undertaken. The mammography revealed micro calcifications on the left breast. A lumpectomy after x-ray identification of these micro calcifications, at the level of the left breast was done. The frozen section histology showed an intraductal carcinoma of high grade with 2 focus, 3mm and 1mm corresponding to Infiltrating left breast carcinoma, grade II tumor of Scarff and Bloom, estrogen and progesterone receptor positive: it wasa multifocal cancer (SBRII, 21 N + / 26, RH +, low Ki 67). A mastectomy of the left breast with ganglion clearing was done immediately. Clearing covered 26 lymph nodes, of which 21 were metastatic and some with capsular rupture. Next, adjuvant chemotherapy type FEC 100 (5-Fluorouracil, Epirubicin, Cyclophosphamide) was undertaken in 6 Cycles. The histological result of the mastectomy specimen confirmed an infiltrating intraductal carcinoma, grade II tumor of Scarff and Bloom. Radiotherapy of ganglion chains of bleeding and chest wall about the mastectomy scar was then carried out over a period of two months. The patient was placed under hormonal therapy for 5 years (Tamoxifen 20 mg per day). Seven months after her presentation at the hospital, dizziness disappeared, her gait became better coordinated. She was being followed up on a regular basis for any new breast pathology.

## Discussion

We have reported a case of Paraneoplastic Cerebellar Syndrome with a secondary discovery of a breast cancer in a 52-year-old patient. Antineuronal antibodies found in the serum and cerebrospinal fluids of this patient were anti-Yo. Questions arising about the presence of antineuronal antibodies in the body of such patient, the cerebellar syndrome and the secondary discovery of a primary gynecological cancer are. Does the paraneoplastic neurologic syndrome always precede the discovery of a cancer? Does the presence of antineuronal antibodies in the serum of a patient imply the presence of a specific cancer? Questions arising about the pathophysiological relationship between these three entities; cerebellar syndrome, antineuronal antibodies, and breast cancer. It will be recalled that the interest of this article lies in the diagnosis of Carcinoma of the breast through the presence of antineuronal antibodies in the serum and cerebrospinal fluid of the patient. There were questions arising about the possible autoimmune nature of paraneoplastic syndrome and the evolution of the syndrome after treatment of cancer. Literature has made several case presentations and responses are according to the authors. Neurological syndromes most often precede the discovery of cancer. Peterson who studied 55 cases of Paraneoplastic cerebellar Syndromes with antibody anti-Yo found that neurological signs precede the discovery of cancer in 65% of cases, with a delay of up to 15 months [3]. Rojas with about 30 patients noted that the paraneoplastic cerebellar syndrome precedes the diagnosis of the tumor in 63% of cases, with an average delay of five months up to 13 months [4]. In our observation the chronology of the diagnosis was as follows: 1) Diagnosis of cerebellar syndrome. 2) Positivity of antineuronal antibodies, highlighted one month after the beginning of hospitalization. 3) Diagnosis of breast cancer. It is difficult to specify in our study the order of installation of these three elements. According to some authors, there are anti-neural autoantibodies in 50% of Cerebellar paraneoplastic syndromes, and it is usually anti-Yo antibodies.

Out of 47 patients who had a Paraneoplastic cerebellar Syndrome, Anderson found an anti-neuronal auto antibody in 23 cases, among which 18 (78%) had an anti-Yo antibody [5]. Shams'ili had described 50 patients suffering from Paraneoplastic cerebellar Syndrome with anti-neuronal antibodies; 38% of the patients had an anti-Yo type of antibody [6]. Dalmau and Posner emphasized two fundamental points: 1) Almost all patients with anti-Yo antibodies in their serum have an associated cancer. 2) This cancer is 90% breast or gynaecological and when a cerebellar syndrome is associated with anti-Yo antibodies, the predictive value of gynecological or breast tumor is close to 100% [7]. Anti-Yo antibodies are positive especially among women and there are rare cases of anti-Yo in a man. Krakauer described a case associated with adenocarcinoma of unknown origin [8]. Meglic also described a case associated with a gastric adenocarcinoma [9]. Different studies have showed that, in breast cancer, several types of antineuronal antibodies may be involved in the pathogenesis of the Paraneoplastic neurologicalsyndromes. Some studies have found as antineuronal antibodies, anti - Hu [10], anti-Ri [11]. We have not found a study showing different antineuronal antibodies in the same patient. According to review of the literature, there are different types of antigens for antineuronal antibodies. Tanaka K, Onodera O. presents the antibody anti-Yo as an antineuronal antibody that reacts with the Antigen, which is a set of two proteins of 32 and 64 kilodaltons in the cytoplasm of the Purkinje cells [12]. Kaneko A, Nishikiori E explained: “From the Western blot, the patient serum has reacted with a 40 - kDa protein” [13]. WirtzPw and SillevisSmitt talked about a case of breast cancer where it was found in the serum, anti-Ri antibodies reacting with antigens nova1 and nova2 at the level of the central nervous system [14]. Sutton Ian, reported case of antineuronal antibodies react with antigens ma2 and antigens Ta in a patient of 58 years having paraneoplastic neurologic syndrome limbicencephalitis [15].

The Paraneoplastic Cerebellar Syndrome would be a model of autoimmune disease of the neurological tissue. A study of the ARTC (Association for Research on brain tumors in Neuro - Oncology) proposes the following hypothesis: … “In other words, fighting his own tumor, the patient attacks his own nervous system as if his body confounded his nervous system with the tumor …” [16]. Nagel A. gave the following hypothesis: the body reacts against the tumor but; against healthy cells in the body (here the Purkinje cells) which have affinities or genetic similarities with the tumor. Cellular immunity or other antigenic determinants are certainly involved, anti-Yo antibodies identified one or more of the human cerebellum Purkinje cell cytoplasm antigens [17]. They were detected and titrated in serum and cerebrospinal fluid by immunohistochemistry or immunofluorescence on sections of cerebellum. In western-blot, anti-Yo reacted against 2 proteins, one of 62KD, the other 34 KD of a cytoplasm of Purkinje cell lysate. The expression of Antigen Yo by a tumor associated with a Paraneoplastic Cerebellar Syndrome and anti-Yo antibody has been demonstratedby Furneaux HM [18]. Anti-Yo anti-bodies of a patient attach to the histological sections of his own tumor, and in certain cases, when the patient died, and an autopsy was performed, it is on sections of his own cerebellum [19, 20]. There are many arguments to consider the Paraneoplastic Cerebellar Syndrome as a neurological autoimmune disease triggered by the tumor expression of neural neo-antigens [21]. If the treatment of the tumor is necessary, various authors have also tried various treatments to inhibit or modulate the immune response. Generally, surgical or medical treatment of the tumor does not improve the neurological signs [22]. Treatments are ineffective or may allow for only discrete and transient improvements. However the discovery of a paraneoplastic syndrome in a patient allows an effective treatment of the tumor with which it is associate [23]. It is difficult to accurately assess the prognosis of gynecological or breast tumors associated with aParaneoplastic Cerebellar Syndrome with anti-Yo antibodies. Although neurological signs lead in more than half of the cases to the discovery of the cancer, the tumors are often already advanced with neurological manifestations. In our study, the patient presented with an important invasion of the axillary lymph nodes, while there were micro-calcifications on mammography and clinical she was well.

## Conclusion

Breast cancer is not always associated with a Paraneoplastic neurological Syndrome and all the cerebellar cases are not paraneoplastic syndromes. Paraneoplastic cerebellar Syndrome precedes the discovery of cancer in 65% of cases. Confronted with a cerebellar clinical picture, we must evoke a Paraneoplastic cerebellar Syndrome and find tumor aetiology. The research of anti-neuronal auto antibodies that are positive in the serum and or cerebrospinal fluid once in Paraneoplastic cerebellar syndrome allows a focused etiological investigation. Anti-Yo auto antibodies are quasi-specific for gynecological or breast tumors. Publications in the literature on cancer and Paraneoplastic cerebellar syndrome have been made for about twenty years. Several hypotheses have been advanced on the pathophysiology and one wonders if someday, we would be able to do very early diagnosis of cancer including the breast cancer on the basis of the antigen-antibody reaction.
